# Associations between monitor-independent movement summary (MIMS) and fall risk appraisal combining fear of falling and physiological fall risk in community-dwelling older adults

**DOI:** 10.3389/fragi.2024.1284694

**Published:** 2024-04-09

**Authors:** Renoa Choudhury, Joon-Hyuk Park, Chitra Banarjee, Miguel Grisales Coca, David H. Fukuda, Rui Xie, Jeffrey R. Stout, Ladda Thiamwong

**Affiliations:** ^1^ Department of Mechanical Engineering, University of Central Florida, Orlando, FL, United States; ^2^ Disability, Aging and Technology Cluster, University of Central Florida, Orlando, FL, United States; ^3^ College of Medicine, University of Central Florida, Orlando, FL, United States; ^4^ School of Kinesiology and Rehabilitation Sciences, College of Health Professions and Sciences, University of Central Florida, Orlando, FL, United States; ^5^ Department of Statistics and Data Science, University of Central Florida, Orlando, FL, United States; ^6^ College of Nursing, University of Central Florida, Orlando, FL, United States

**Keywords:** falls, physical activity, accelerometry, aging, fear of falling, fall risk, MIMS

## Abstract

**Introduction:** Fall Risk Appraisal (FRA), a process that integrates perceived and objective fall risk measures, serves as a crucial component for understanding the incongruence between fear of falling (FOF) and physiological fall risk in older adults. Despite its importance, scant research has been undertaken to investigate how habitual physical activity (PA) levels, quantified in Monitor-Independent Movement Summary (MIMS), vary across FRA categories. MIMS is a device-independent acceleration summary metric that helps standardize data analysis across studies by accounting for discrepancies in raw data among research-grade and consumer devices.

**Objective:** This cross-sectional study explores the associations between MIMS (volume and intensity) and FRA in a sample of older adults in the United States.

**Methods:** We assessed FOF (Short Falls Efficacy Scale-International), physiological fall risk (balance: BTrackS Balance, leg strength: 30-s sit-to-stand test) and 7-day free-living PA (ActiGraph GT9X) in 178 community-dwelling older adults. PA volume was summarized as average daily MIMS (MIMS/day). PA intensity was calculated as peak 30-min MIMS (average of highest 30 non-consecutive MIMS minutes/day), representing a PA index of higher-intensity epochs. FRA categorized participants into following four groups: Rational (low FOF-low physiological fall risk), Irrational (high FOF-low physiological fall risk), Incongruent (low FOF-high physiological fall risk) and Congruent (high FOF-high physiological fall risk).

**Results:** Compared to rational group, average MIMS/day and peak 30-min MIMS were, respectively, 15.8% (*p* = .025) and 14.0% (*p* = .004) lower in irrational group, and 16.6% (*p* = .013) and 17.5% (*p* < .001) lower in congruent group. No significant differences were detected between incongruent and rational groups. Multiple regression analyses showed that, after adjusting for age, gender, and BMI (reference: rational), only irrational FRA was significantly associated with lower PA volume (β = −1,452.8 MIMS/day, *p* = .034); whereas irrational and congruent FRAs were significantly associated with lower “peak PA intensity” (irrational: β = −5.40 MIMS/day, *p* = .007; congruent: β = −5.43 MIMS/day, *p* = .004).

**Conclusion:** These findings highlight that FOF is a significant barrier for older adults to participate in high-intensity PA, regardless of their balance and strength. Therefore, PA programs for older adults should develop tailored intervention strategies (cognitive reframing, balance and strength exercises, or both) based on an individual’s FOF and physiological fall risk.

## Introduction

In the United States (US), over 14 million adults aged 65 years or older fall each year (Moreland et al., 2020; Kakara et al., 2023). According to the US Centers for Disease Control and Prevention, about 20% of falls in older adults cause serious injuries, which results in limited functional mobility, loss of independence, reduced quality of life, and premature death (Ambrose et al., 2013). Fear of falling (FOF) has been recognized as an important psychological aspect associated with falls in older adults (Jansen et al., 2021). However, studies report that many older adults might show a discrepancy between their FOF and physiological fall risk, known as maladaptive fall risk appraisal (FRA) (Thiamwong et al., 2021a), and such discrepancies can lead to adverse consequences. For example, individuals with low physiological fall risk but high FOF may overestimate their actual fall risk and restrict their daily activities, which can further lead to physical deconditioning and loss of muscle strength (Deshpande et al., 2008). On the contrary, those with high physiological fall risk but low FOF may underestimate their actual fall risk and engage in unnecessary risky behavior beyond their physical capacity, making them even more vulnerable to falling (Delbaere et al., 2010).

**FIGURE 1:**
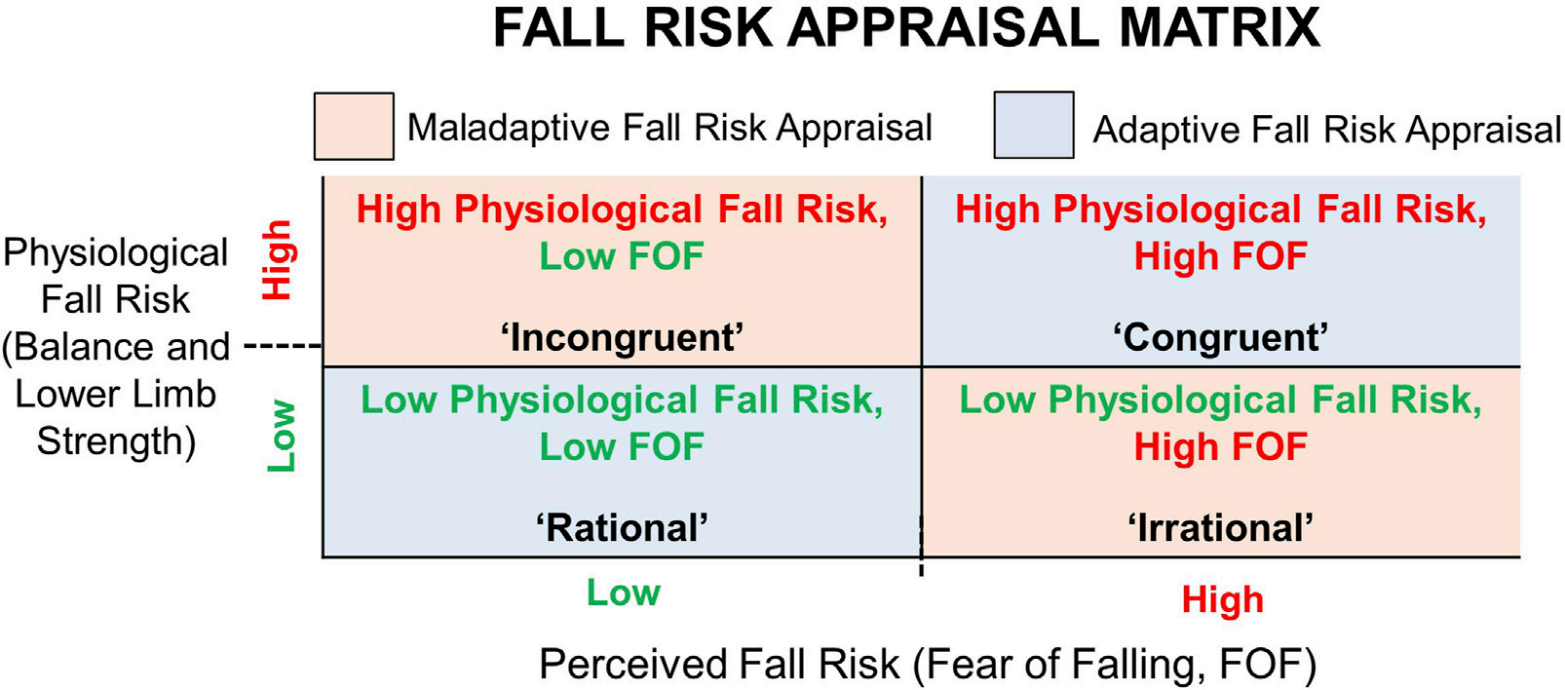
Fall Risk Appraisal (FRA) based on Fear of Falling (FOF) and physiological fall risk. Maladaptive FRA = mismatch between FOF and physiological fall risk; Adaptive FRA = FOF aligned with physiological fall risk.

Therefore, FRA combining subjective and objective fall risk measures is important for understanding the discrepancy between FOF and physiological fall risk in older adults to inform more targeted interventions for fall prevention (Thiamwong et al., 2020a; Thiamwong et al., 2020b). FRA is a two-dimensional fall risk assessment matrix that classifies older adults into four groups based on their FOF and physiological fall risk status (Thiamwong, 2020). In FRA matrix, as shown in Figure 1, two groups have their FOF level aligned with their physiological fall risk status, which are denoted as Rational (low FOF-low physiological fall risk) and Congruent (high FOF-high physiological fall risk). The other two groups show a mismatch between their FOF level and physiological fall risk status and are denoted as Incongruent (low FOF-high physiological fall risk) and Irrational (high FOF-low physiological fall risk).

Prior research has mostly focused on exploring the independent associations of FOF and objective fall risk measures with physical activity (PA) participation in older adults (Gregg et al., 2000; Chan et al., 2007; Zijlstra et al., 2007; Heesch et al., 2008; Mendes da Costa et al., 2012). To date, only a small number of studies have investigated the combined effects of FOF and objective fall risk on PA engagement. For example, one study examined the joint associations of FOF and objective fall risk with everyday walking activities in older adults. This study used a four-group categorization from (Delbaere et al., 2010), and found that the number of steps/day in their study sample was in accordance with objective fall risk rather than FOF (Jansen et al., 2021). Another study examined accelerometry-based PA levels between FRA categories using the intensity cut-point approach and found that participants with high FOF accumulated significantly less time in moderate-to-vigorous PA (MVPA) compared to those with rational FRA, regardless of their balance performance (Thiamwong et al., 2023). However, there exists a lack of evidence on how habitual PA levels, expressed in Monitor-Independent Movement Summary (MIMS) units, differ between FRA categories in older adults.

MIMS is used to summarize the acceleration measurements obtained on the x-, y-, and z-axes of wrist-worn activity monitors. This PA metric was first introduced in 2019 to summarize participant-level PA data for the 2011-2012 and 2013-2014 cycles of the US National Health and Nutrition Examination Survey (NHANES) (John et al., 2019). The major benefit of using MIMS is that it is generated by a nonproprietary device–independent universal algorithm, allowing us to compare the total movement across studies regardless of the heterogeneity introduced by different brands, models and device types (such as consumer vs. research-grade) (John et al., 2019). Similar to other traditional PA metrics such as steps/day or daily activity counts, PA volume can be expressed as daily MIMS (i.e., total MIMS unit accumulated per day) across valid days of assessment, where larger MIMS/day indicates higher daily PA volume (Wolff-Hughes et al., 2014).

Traditionally, quantification of accelerometer-measured PA intensity has been predominantly based on minutes/day (or minutes/week) spent in MVPA, using either manufacturer-specific or device-specific cut points corresponding with ≥3 Metabolic Equivalents of Task (METs) (Troiano et al., 2008). Recently, to establish an intensity-based expression for MIMS units, the concept of peak 30-min MIMS has been introduced (Zheng et al., 2023). It is analogous to the concept of peak 30-min cadence, i.e., the average of 30 highest cadence (steps/minutes) values within a day, representing an individual’s best efforts (Tudor-Locke et al., 2012). Similar to cadence (steps/minutes), MIMS/minutes values were shown to have a strong correlation with higher PA intensity (John et al., 2019). Therefore, peak 30-min MIMS (i.e., the average of the highest 30 non-consecutive MIMS [minutes/day] values within a day) can be used as a measure of higher-intensity epochs across the PA monitoring period (Zheng et al., 2023). Evaluating daily MIMS (volume) and peak 30-min MIMS (intensity) can facilitate a more comprehensive assessment of PA and its relationship with FRA.

Thus, the aim of this study is to investigate the associations between wrist-worn accelerometer-measured PA (expressed as daily MIMS and peak 30-min MIMS) and FRA in a sample of community-dwelling older adults. We are particularly interested in the question: “Which of the maladaptive FRA groups, i.e., Incongruent (low FOF-high physiological fall risk) and Irrational (high FOF-low physiological fall risk), differ more from the Rational (low FOF-low physiological fall risk) group in terms of habitual PA level?.” This will allow us to understand which of the two factors—FOF or physiological fall risk—has a stronger relationship with reduced PA participation among older adults.

## Materials and methods

### Study design and participants

In this cross-sectional study, purposive sampling was used to recruit 178 community-dwelling older adults from the region of Central Florida, United States, between February 2021 and March 2023. The inclusion criteria were: i) 60 years of age or older; ii) being able to walk with or without an assistive device (but without the assistance of another person); iii) no marked cognitive impairment [i.e., Memory Impairment Screen score ≥5 (Buschke et al., 1999)], iv) fluency in English or Spanish, and v) living in their own homes or apartments. The exclusion criteria were: i) medical conditions that prevent PA engagement (e.g., shortness of breath, tightness in the chest, dizziness, or unusual fatigue at light exertion), ii) unable to stand on the balance plate, iii) currently receiving treatment from a rehabilitation facility, and iv) having medical implants (e.g., pacemakers). This study was approved by the Institutional Review Board at the University of Central Florida (Protocol No: 2189; 10 September 2020). All subjects provided written informed consent to participate. This cross-sectional assessment required one visit to the study site during which participants completed a demographic survey and anthropometric measurements, followed by assessments of FOF and physiological fall risk. At the end of the visit, each participant was fitted with a wrist-worn accelerometer for 7-day PA monitoring in free-living conditions.

### Measurements

#### Fear of falling (FOF)

FOF was assessed using the Short Falls Efficacy Scale-International (FES-I) questionnaire (Yardley et al., 2005; Kempen et al., 2008). It is a 7-item, self-administered tool that uses a 4-point Likert scale to measure the level of concern about falling while performing seven activities (1 = not at all concerned to 4 = very concerned). The total scores ranged from 7 to 28. Short FES-I scores of 7–10 indicated low FOF, while scores of 11–28 indicated high FOF.

### Physiological fall risk

Physiological fall risk was assessed using balance test and lower limb strength assessment. BTrackS Balance System (Balance Tracking Systems, San Diego, CA, United States) was used to measure static balance. This system includes a portable BTrackS Balance Plate and BTrackS Assess Balance Software running on a computer. It has shown high test–retest reliability (intraclass correlation coefficient, ICC = 0.83) and excellent validity (Pearson’s product-moment correlations, r > 0.90) in evaluating static balance (Levy et al., 2018). The test protocol included four trials (each trial taking 20 s) with less than 10 s of inter-trial delays. During the trials, participants were asked to stand still on the BTrackS Balance Plate with their eyes closed, hands on their hips, and feet placed shoulder-width apart. BTrackS balance plate is an FDA-registered, lightweight force plate that measures center of pressure (COP) excursions during the static stance. The first trial was done for familiarization only. Results from the remaining three trials were used to calculate the average COP path length (in cm) across trials. COP path length is considered as a proxy measure for postural sway magnitude; thus, the larger the COP path length, the greater the postural sway is (Goble et al., 2017). COP path length of 0–30 *cm* was used to indicate normal balance, while ≥31 *cm* indicated poor balance (Thiamwong et al., 2021b).

Lower limb strength was assessed using the 30-s sit-to-stand (STS) test, in accordance with the established protocol (Yee et al., 2021; Choudhury et al., 2023). Participants were instructed to keep their arms folded across their chest, rise from a seated position on a chair to a standing posture and return to the sitting position as many times as possible within 30 s. The number of chair stands completed was counted and recorded. If a participant used his/her arms to stand, the test was stopped, and the score was recorded as zero. Age- and gender-specific STS normative scores were used as cut-offs to classify participants into below-average and average STS scores, as shown in Table 1 (Rikli and Jones, 1999). A below-average STS score was indicative of a higher risk of fall. Meeting both normal balance and average STS score criteria was defined as low physiological fall risk, while not meeting either or both criteria was defined as high physiological fall risk.

**TABLE 1:** Age and gender-specific below average scores for 30-s sit-to-stand test.

Age group	Gender	
	Women	Men
60–64 years	<12	<14
65–69 years	<11	<12
70–74 years	<10	<12
75–79 years	<10	<11
80–84 years	<9	<10
85–89 years	<8	<8
90–94 years	<4	<7

### Fall risk appraisal (FRA) matrix

The FRA matrix was obtained using a combination of FOF and physiological fall risk status. Participants were grouped into the following four categories based on their FOF and physiological fall risk according to existing literature (Thiamwong et al., 2020a): i) Rational (low FOF-low physiological fall risk), ii) Irrational (high FOF-low physiological fall risk), iii) Incongruent (low FOF-high physiological fall risk), and iv) Congruent (high FOF-high physiological fall risk).

#### Physical activity (PA)

PA was assessed using ActiGraph GT9X Link (ActiGraph LLC., Pensacola, FL, United States), which contains a triaxial accelerometer with a dynamic range of ±8 gravitational units (g). The device was initialized to record acceleration data at 30 *Hz* sampling frequency. Participants wore the ActiGraph on their non-dominant wrists for seven consecutive days in free-living conditions. They were given instructions to wear it during waking hours and remove it only during sleeping, showering, swimming and medical imaging tests. After 7-day of PA monitoring, ActiGraph devices were collected from participants. Participants with ≥4 valid days were included in the analysis, and a day was considered valid if participants wore the device for at least 14 h or more (Choudhury et al., 2023).

Raw acceleration data were downloaded as “.csv” files using ActiLife software v6.13.4 (ActiGraph LLC, Pensacola, FL, United States) and converted into MIMS units using MIMSunit package (John et al., 2019) in R statistical software (R Core Team, Vienna, Austria). The data processing steps included: i) interpolating data to a consistent sampling rate (i.e., 100 *Hz*) to account for inter-device variability in sampling rate, ii) extrapolating data to extend maxed-out signals to account for inter-device variability in dynamic range, iii) band-pass filtering to remove artifacts from acceleration signals that do not pertain to voluntary human movement, and iv) aggregation of processed signals from each axis into a sum of MIMS-units that represents the total amount of movement activity [details on MIMS-unit algorithm are published elsewhere (John et al., 2019)].

PA volume, denoted by daily MIMS (MIMS/day), was calculated by summing up all triaxial MIMS/minutes accumulated throughout a day and averaged across all valid days. PA intensity, expressed as peak 30-min MIMS, was obtained by (a) first rank ordering a participant’s triaxial MIMS/minutes values within each valid day, (b) calculating the average of the highest 30 MIMS/minutes values within each day, and (c) finally taking the average of the resulting MIMS/minutes values across all valid wear days.

#### Anthropometric measurements

Height (in cm) was measured using a stadiometer. Body mass (in kilograms) was measured using a digital scale with no shoes. Body mass index (BMI) was calculated as the weight (kg) divided by the square of height (m^2^).

### Statistical analyses

All statistical analyses were performed in R statistical software (version 4.1.2, R Core Team, Vienna, Austria) with statistical significance level set at .05. Descriptive characteristics of participants were summarized as mean (standard deviation, SD) for normally distributed continuous variables, as median (Interquartile Range, IQR) for non-normally distributed continuous variables, and as frequency (percentage) for categorical variables, stratified by FRA categories. The Shapiro-Wilk test was performed to check if a continuous variable followed a normal distribution. Differences across groups were examined using one-way analysis of variance (ANOVA) for normally distributed data and Kruskal–Wallis test for non-normally distributed data, with Bonferroni adjustment for *post hoc* comparisons.

Multiple linear regression was conducted for each outcome variable (i.e., daily MIMS and peak 30-min MIMS) using the four FRA groups—“Rational,” “Irrational,” “Incongruent” and “Congruent”—as explanatory variables, controlled by age, gender and BMI. *A priori* sample size calculation for multiple linear regression revealed that the minimum number of samples for 8 explanatory variables at a statistical power level of 0.8, α = 0.05, and a medium effect size (Cohen *f* ^*2*^ = 0.15) would be 108; therefore, our sample size (i.e., *N* = 178) had sufficient statistical power for multiple regression. The rational group (i.e., low FOF-low physiological fall risk) was selected as the reference group in the regression analysis.

## Results

**FIGURE 2:**
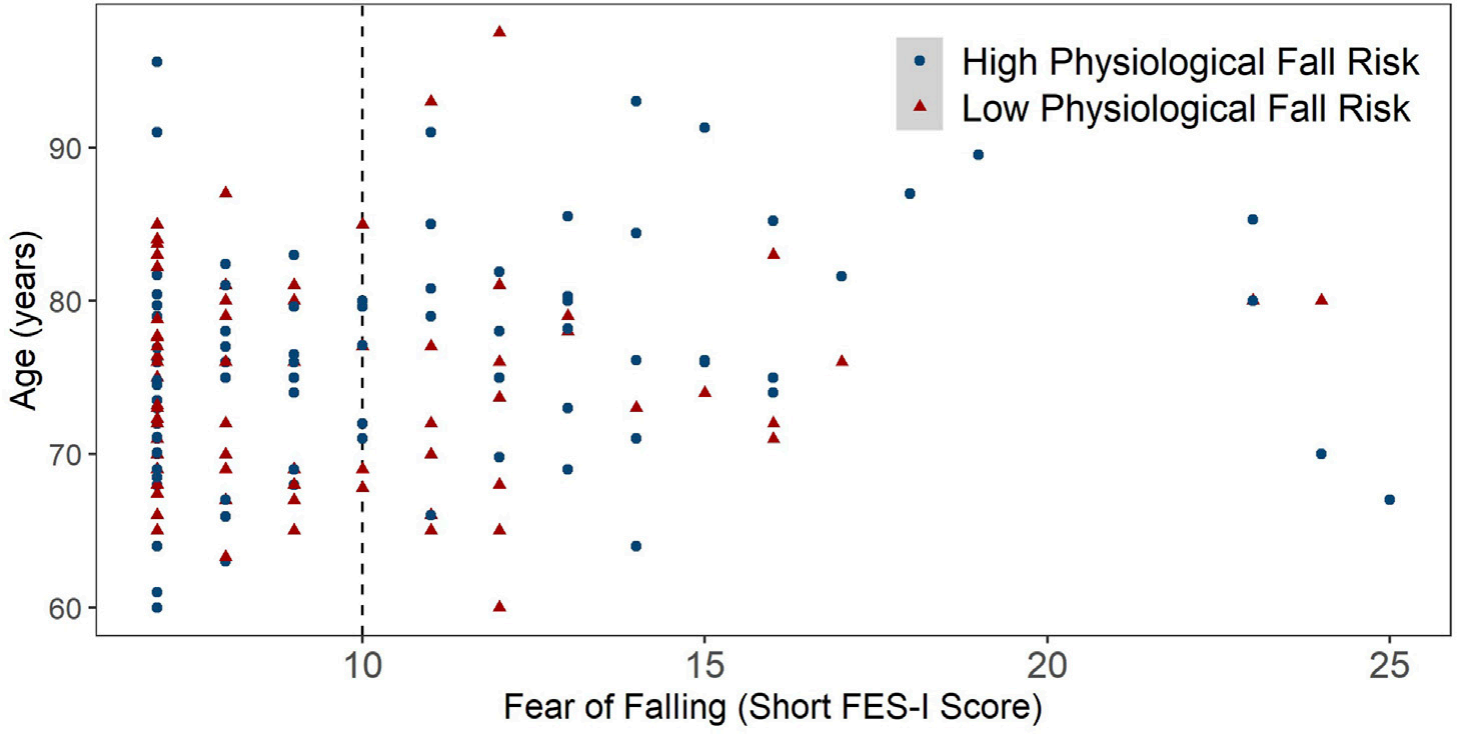
Scatterplot of Age (years) across Fear of Falling scores, stratified by physiological fall risk status. Low physiological fall risk = meeting both normal static balance cut-off and average sit-to-stand score cut-off. High physiological fall risk = not meeting normal static balance cut-off or average sit-to-stand score cut-off or both.

Among 178 participants, 163 samples were included in the analyses, after retaining only those who had at least 4 days of valid PA data and completed both FOF and physiological fall risk assessments. The mean (SD) age of participants was 75.3 (7.1) years, and 73.6% of participants were in 60–79 years of age group (*n* = 120) and 26.4% were above 80 years of age (*n* = 43). Figure 2 shows the scatterplot of participants’ age (years) and FOF scores, stratified by physiological fall risk status. The proportion of participants with low FOF was 71.7% (*n* = 86) in the 60–79 years of age group and 48.8% (*n* = 21) in the ≥80 years of age group. The median (IQR) BMI of participants was 26.6 (6.3) kg/m^2^ and majority of participants were female (79.1%). The median (IQR) Short FES-I score was 9 (5) and 34.4% of participants had high FOF. The median (IQR) COP path length was 27 (15) *cm*, and the median (IQR) sit-to-stand score was 13 (6) reps. 38.0% of participants had poor balance, 27.0% had below average lower limb strength, and 48.5% showed both poor balance and below average lower limb strength. Finally, 37.4% of participants were screened as rational (*n* = 61), 14.2% were irrational (*n* = 23), 28.2% were incongruent (*n* = 46) and 20.2% were congruent (*n* = 37). Table 2 summarizes the characteristics of study participants according to FRA categories.

**TABLE 2:** Participant characteristics stratified by Fall Risk Appraisal matrix.

Variables	Overall (*N* = 163)	Fall risk appraisal groups				
		Rational (*n* = 61, 37.4%)	Irrational (*n* = 23, 14.2%)	Incongruent (*n* = 46, 28.2%)	Congruent (*n* = 33, 20.2%)	*p*-value
Age, years, mean (SD)	75.3 (7.1)	74.3 (5.8)	75.2 (8.6)	74.3 (7.0)	78.8 (7.6)	**.019** ^a^
Gender, female, *n* (%)^b^	129 (79.1%)	47 (77.0%)	21 (91.3%)	33 (71.8%)	28 (84.8%)	.221
BMI, kg/m^2^, median (IQR)	26.6 (6.3)	24.9 (6.4)	28.1 (6.2)	26.9 (4.7)	28.9 (5.8)	**.007** ^a^
Fear of falling, Short FES-I score, median (IQR)	9 (5)	7 (1)	12 (4)	7.5 (2)	14 (4)	**<.001**
Static balance, COP path length (cm), median (IQR)	27 (15)	21 (7)	23 (10.5)	34.5 (10.2)	42 (18)	**<.001**
Dynamic balance, sit-to-stand score (reps), median (IQR)	13 (6)	15 (4)	14 (7)	10 (6.8)	8 (7)	**<.001**
Daily MIMS, MIMS/day, mean (SD)	9,543 (2,891)	10,408 (2,439)	8,760 (3,016)	9,411 (2,749)	8,677 (3,408)	**.016** ^b^
Peak 30-min MIMS per day, MIMS/day, mean (SD)	36.9 (8.5)	39.9 (8.3)	34.3 (8.2)	37.2 (7.9)	32.9 (7.9)	**.001** ^b^

*p-value was calculated using Pearson’s Chi-squared test.

Bold indicates statistically significant results.

Bonferroni’s post-hoc result showed that:

^a^ Average age and BMI in congruent group were significantly higher than rational group.

^b^ Average daily MIMS and peak 30-min MIMS in irrational and congruent groups were significantly lower than rational group.

**FIGURE 3:**
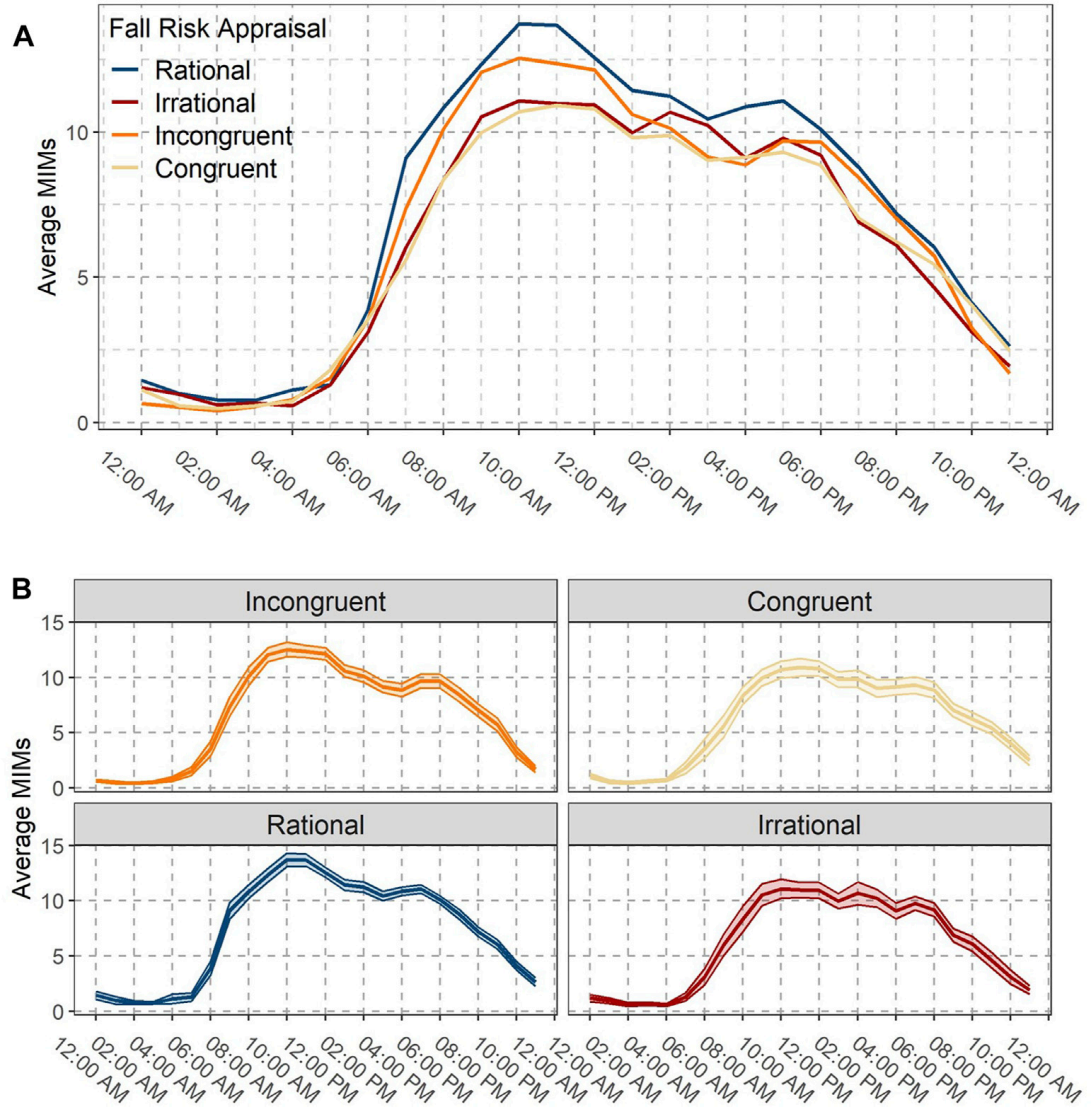
**(A)** Daily patterns of average MIMS per hour by Fall Risk Appraisal (FRA) groups. **(B)** Mean (line) and standard error (shaded area) of MIMS per hour for each FRA group.

In Figure 3A, the variations in average MIMS (MIMS/hours) over 24-h by FRA categories are shown. The average MIMS across all groups was in general low at night, then substantially increased during morning hours and gradually decreased as the day progressed and evening approached. In Figure 3B, the mean (line) and standard error (shaded area) of MIMS/hours for each FRA group is shown. Overall, rational group showed the highest average MIMS/hours across the day hours, while congruent had the lowest average MIMS/hours. Among maladaptive FRA groups, the peak was higher in incongruent group than their irrational counterparts, which indicates the potential role of FOF in limiting high-intensity PA participation.

The mean (SD) age in congruent group was 78.8 (7.6) years, which was higher than both rational (74.3 [5.8] years, *p* = .005) and incongruent (74.3 [7.0] years, *p* = .010) groups, as shown in Supplementary Figure S1. This suggests that prevalence of high FOF, irrespective of balance performance and lower limb strength, may increase with advanced age. Also, the median (IQR) BMI in congruent group (28.9 [5.8]) kg/m^2^) was higher in comparison to rational (24.9 [6.4] kg/m^2^, *p* = .001) and incongruent (26.9 [4.7] kg/m^2^, *p* = .018) groups (shown in Supplementary Figure S2), indicating that higher BMI in older adults may result in high FOF. However, no significant group differences were observed between rational and irrational groups in terms of age and BMI.

**FIGURE 4:**
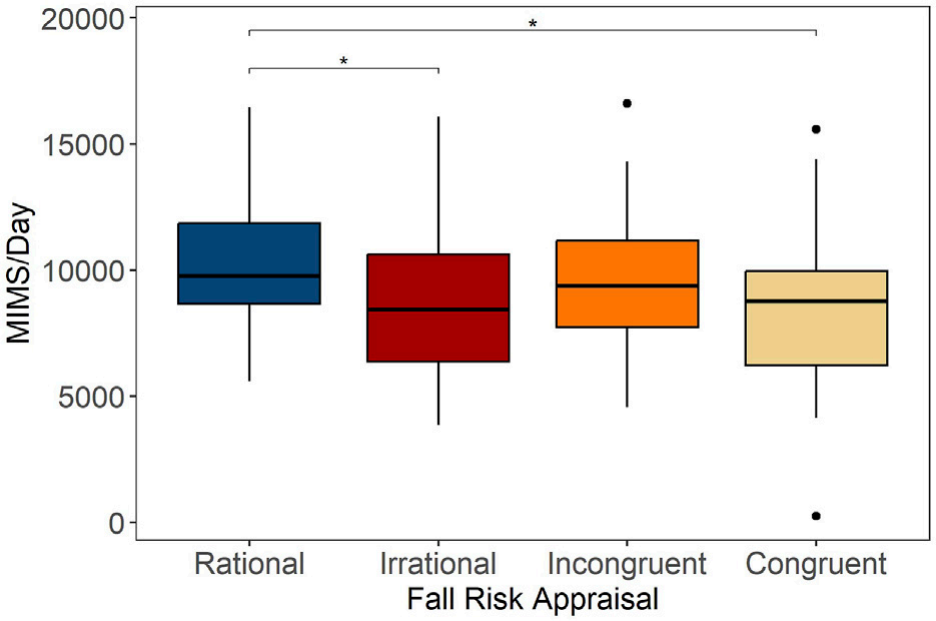
Average daily MIMS (MIMS/day) across categories of Fall Risk Appraisal combining FOF and physiological fall risk, *p < .05.

**FIGURE 5:**
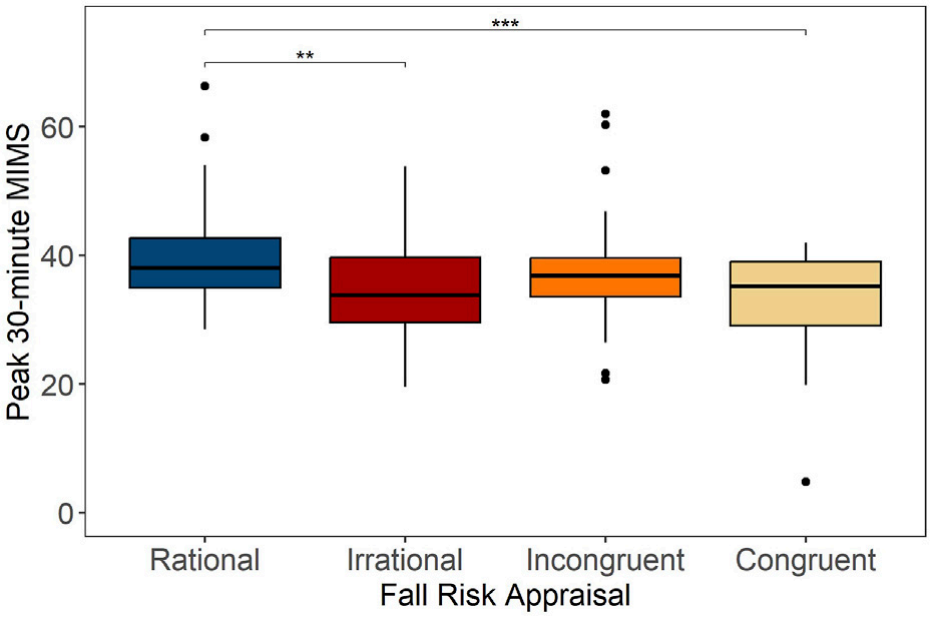
Peak 30-min MIMS per day across categories of Fall Risk Appraisal combining FOF and physiological fall risk, **p < .01, ***p < .001.

The mean (SD) daily MIMS in rational group was 10,408 (2,439) MIMS/day, which was 15.8% higher than irrational (*p* = .025) and 16.6% higher than congruent (*p* = .013) groups, as shown in Figure 4. Also, the mean (SD) peak 30-min MIMS in rational group was 39.9 (8.3) MIMS/day, which was 14.0% higher than irrational (*p* = .004) and 17.5% higher than congruent (*p* < .001) groups (Figure 5). Compared to rational group, incongruent participants showed no significant differences in PA volume and intensity, despite having poor balance and below average lower limb strength.

Table 2 presents the regression models for daily MIMS. In comparison to reference group (i.e., rational), lower PA volume was associated with irrational (*β [SE]* = −1,463.2 [687.7] MIMS/day, *p* = .035) and congruent (*β [SE]* = −1,579.5 [582.9] MIMS/day, *p* = .007) FRAs in Model 1 (unadjusted). In model 2, after adjusting for age, gender and BMI, only irrational FRA was significantly associated with lower PA volume (*β [SE]* = −1,476.41 [582.26] MIMS/day, *p* = .025; regression coefficients of covariates are presented in Supplementary Table S1).

Results for regression analysis for peak 30-min MIMS are presented in Table 3. In model 1 (unadjusted), lower ‘peak PA intensity’ was associated with irrational (*β [SE]* = −5.63 [1.99] MIMS/day, *p* = .005) and congruent FRAs (*β [SE]* = −7.06 [1.76] MIMS/day, *p* < .001) compared to the reference group. In Model 2 (Table 4), after adjusting for age, gender and BMI, both irrational and congruent FRAs were still significantly associated with lower “peak PA intensity”(irrational: *β [SE]* = −5.40 [1.97] MIMS/day, *p* = .007; congruent: *β [SE]* = −5.43 [1.86] MIMS/day, *p* = .004; regression coefficients of covariates are presented in Supplementary Table S2).

**TABLE 3:** Association between Fall Risk Appraisal groups and average daily MIMS (MIMS/day) using linear regression. Model 2 was adjusted for age, gender and BMI.

Daily MIMS, MIMS/day (reference: Rational)	Model 1 (unadjusted)		Model 2 (adjusted)	
	*B (SE)*	*p*	*β (SE)*	*p*
Irrational	−1,648.2 (691.2)	**.018**	−1,603.1 (686.4)	**.021**
Incongruent	−997.3 (551.6)	.072	−883.9 (539.9)	.104
Congruent	−1731.4 (610.4)	**.005**	−1,206.6 (647.7)	.064

Bold indicates statistically significant results.

**TABLE 4:** Association between Fall Risk Appraisal groups and peak 30-min MIMS per day (MIMS/day) using linear regression. Model 2 was adjusted for age, gender and BMI.

Peak 30-min MIMS per day, MIMS/day (reference: Rational)	Model 1 (unadjusted)		Model 2 (adjusted)	
	*B (SE)*	*p*	*β (SE)*	*p*
Irrational	−5.63 (1.99)	**.005**	−5.40 (1.97)	**.007**
Incongruent	−2.76 (1.59)	.084	−2.52 (1.55)	.106
Congruent	−7.06 (1.76)	**<.001**	−5.43 (1.86)	**.004**

Bold indicates statistically significant results.

## Discussion

This is the first study, to our knowledge, to evaluate the associations of FRA with daily MIMS and peak 30-min MIMS in a sample of community-dwelling US older adults. In general, both the volume and intensity of PA were highest in the rational group and lowest in the congruent group. In maladaptive FRA groups, high FOF (i.e., irrational FRA) was associated with lower PA volume and intensity compared to the reference group (i.e., rational FRA), but no significant differences were observed for high physiological fall risk (i.e., incongruent FRA).

Prior research has shown that FOF is associated with reduced PA levels in community-dwelling older adults using objectively measured PA data (Jefferis et al., 2014; Choudhury et al., 2022). Our results broadly agree with it, showing that total daily PA volume was significantly lower in two high FOF groups (i.e., irrational and congruent) than the rational group. This suggests that regardless of balance performance and lower limb strength, low FOF was associated with high PA volume in our study sample. In linear regression analysis, after accounting for age, gender and BMI, reduced daily MIMS was significantly associated with irrational FRA, but not with congruent FRA. It can be attributed to the fact that the average age and BMI of congruent participants were higher than all other groups, and evidence suggests that that increasing older age and higher BMI contribute to lower PA levels in older adults (Smith et al., 2015).

We did not observe any significant difference between two low FOF groups (i.e., rational and incongruent) in terms of daily PA volume. This suggests that, for maladaptive FRA, high physiological fall risk (not high FOF) had a stronger association with reduced daily PA accumulation in our study sample. In contrast to our findings, a recent study found that low physiological fall risk was more strongly associated with increased walking activity (steps/day) than low perceived fall risk in a sample of community-dwelling German older adults (*n* = 294) (Jansen et al., 2021). However, it should be noted that Jansen et al. used multiple independent risk factors (i.e., previous falls, balance impairment, gait impairment, and multimedication) to distinguish between high and low physiological fall risk, whereas they used only one tool (Short FES-I) to assess perceived fall risk. Furthermore, participants with low FOF and high physiological fall risk in that German older adult cohort (Jansen et al., 2021) were relatively older than those in our study sample [mean (SD) age: 81.6 (5.5) years vs. 74.3 (7.0) years in our study]. Previous studies indicate that the likelihood of reduced participation in PA gradually increases with advanced age, because of age-related declines in muscle mass, muscle strength, and functional fitness (i.e., the physical capacity to perform activities of daily living independently and without the early onset of fatigue) (Milanović et al., 2013; Westerterp, 2018; Suryadinata et al., 2020). Therefore, future research should examine how age-related functional declines mediate the relationship between maladaptive FRA and daily PA volume in older adults.

In our study, the peak PA intensity in both high FOF groups (i.e., irrational and congruent) was significantly lower than the rational group. Despite the differences in the PA metrics, this is in general agreement with the previous findings that showed older adults with irrational and congruent FRAs were more likely to spend less time in MVPA (Thiamwong et al., 2023). Interestingly, after adjusting for confounders, the decrease in peak 30-min MIMS for irrational and congruent groups was almost equivalent in our study. This suggests that older adults with high FOF may restrict their participation in high intensity PA, irrespective of their physiological fall risk status. Our findings extend the previously reported association between PA intensity and FOF in older adults (Sawa et al., 2020), highlighting the need to integrate cognitive behavioral therapy to reduce FOF in fall intervention programs.

For peak 30-min MIMS, we did not find any significant difference between two low FOF groups (i.e., rational and incongruent). This suggests that, similar to total PA volume, peak PA intensity was more strongly associated with high FOF (rather than high physiological fall risk) for maladaptive FRA in our sample. Unlike MVPA cut points that exclude PA intensities ≤3 METs or equivalent, peak 30-min MIMS considers acceleration magnitudes ranging from lower to higher peak efforts within a day, enabling comparison over the whole spectrum of PA intensity levels (e.g., light vs. vigorous) (Zheng et al., 2023). Further research should investigate domains of peak PA efforts across different FRA groups, so that informed strategies can be developed to promote high-intensity PA participation according to the perceived and physiological risk of fall.

Based on the findings of our study, it can be conferred that the FRA assessment may be useful in designing customized PA interventions to promote an active lifestyle in older adults. For example, to increase PA participation in older adults with irrational FRA, cognitive behavioral therapy can be integrated into PA programs to improve their self-efficacy and sense of control over falling (Tennstedt et al., 1998). For incongruent FRA, PA recommendations should include exercise regimens specifically designed to reduce physiological fall risk, such as high intensity balance and strength training, in addition to aerobic activities (Sherrington et al., 2008). On the other hand, older adults with congruent FRA may benefit from PA programs that combine both balance and strength exercises, and cognitive behavioral therapy (Brouwer et al., 2003).

A strength of our study is the use of MIMS metric to provide a comprehensive PA assessment (volume and intensity) enabling reliable, cross-study comparisons of our findings with other MIMS-based studies regardless of the device type, model or manufacturer. Furthermore, we used evidence-based cut-off points to determine FOF level (low vs. high FOF), balance status (poor vs. normal balance) and lower limb strength (below average vs. average strength) to categorize participants into FRA groups. However, our study has several limitations. First, to determine physiological fall risk status, we didn’t use the Physiological Profile Assessment (Delbaere et al., 2010) or multiple independent risk factors (Jansen et al., 2021), which might have led to different group formations than those studies. Instead, we used static balance and lower limb strength as physiological fall risk indicators. While balance and strength deficits are important predictors of falls in older adults, they might not account for all aspects of physiological fall risk (such as gait impairment, visual and sensory deficits, use of multi-medications etc.) (Fabre et al., 2010). Second, it is to be noted that the balance performance measure (i.e., static balance) used in this study may not capture the full spectrum of an individual’s balance capabilities. There are different measures of balance performance, including static steady-state balance (i.e., the ability to maintain a steady position while standing or sitting), dynamic steady-state balance (i.e., the ability to maintain a steady position while performing postural transitions and walking), proactive balance (i.e., the ability to anticipate and mitigate a predicted postural disturbance), and reactive balance (i.e., the ability to recover a stable position following an unexpected postural disturbance) (Shumway-Cook and Woollacott, 2007). Therefore, future studies may consider using more comprehensive assessments of balance performance in older adults to define physiological fall risk in FRA. Third, our study only considered FOF as the psychological fall risk measure in FRA and did not investigate other psychological constructs such as falls efficacy or balance confidence (Moore et al., 2011). FOF and falls efficacy are two major fall-related psychological constructs in preventing and managing fall risks in older adults. It is to be noted that, though FOF and falls efficacy are correlated, they represent theoretically distinct concepts (Hadjistavropoulos et al., 2011). FOF is defined as “the lasting concerns about falling that leads to an individual avoiding activities that one remains capable of performing.” Some common instruments for FOF measurement include FES-I, Short FES-I, Iconographical Falls Efficacy Scale (ICON-FES), Geriatric Fear of Falling Measure (GFFM), Survey of Activities and Fear of Falling in the Elderly (SAFE), Fear of Falling Avoidance Behaviour Questionnaire (FFABQ) etc., (Soh et al., 2021). On the other hand, falls efficacy is defined as the perceived confidence in one’s ability to carry out activities of daily living without experiencing a fall (Moore and Ellis, 2008). Existing instruments for measuring falls efficacy include Falls Efficacy Scale (FES), modified FES (MFES), Perceived Ability to Prevent and Manage Fall Risks (PAPMFR), and Perceived Ability to Manage Risk of Falls or Actual Falls (PAMF) (Soh et al., 2021). Prior research has reported that, compared to FOF, falls efficacy shows stronger relationship with measures of basic and instrumental activities of daily living (ADL-IADL), and physical and social functioning (Tinetti et al., 1994). Therefore, future studies should consider exploring the combined effects of falls efficacy and physiological fall risk measures on habitual PA level to determine whether FOF or fall efficacy should be considered as a target for PA interventions in older adults. Fourth, to date, there exists no established cut-offs for the MIMS metric to categorize total PA volume and intensity that correspond to meeting national PA guidelines, and it is still unknown how well MIMS/minute can estimate energy expenditure (Vilar-Gomez et al., 2023). Our study just provided a first step toward the use of a standardized metric to associate PA behavior with FRA in a community-dwelling older adult sample in the US. Future studies should examine such associations in large, nationally representative populations to establish benchmark values for daily MIMS and peak 30-min MIMS in different FRA categories. Fifth, the cross-sectional design of the study didn’t allow us to determine a causal relationship between FRA and PA, so reverse and/or bidirectional causality might still be present. Sixth, although we controlled for age, gender, and BMI in the regression analyses, there remains the possibility of additional residual confounding [such as neuropsychological constructs that have been associated with FOF, which include depression, anxiety, neuroticism, attention, and executive function (Delbaere et al., 2010)]. Finally, our sample size was relatively small and 79% of participants were female. The generalizability of our findings might be restricted by the small, female dominant nature of our sample.

In conclusion, compared to rational FRA, the habitual PA level (daily MIMS and peak 30-min MIMS) was lower in both high FOF groups (i.e., irrational and congruent), but not in incongruent group. This suggests that, for maladaptive FRA in our study sample, high perceived fall risk had a stronger association with reduced PA level, rather than high physiological fall risk. When controlled for covariates, decrease in peak PA intensity remained significantly associated with irrational and congruent FRAs, indicating that older adults with high FOF performed PA at lower peak efforts, irrespective of their physiological fall status. Future prospective studies should focus on identifying the optimal habitual PA level (total PA volume and peak PA intensity) in accordance with an older adult’s FOF and physiological fall risk to better inform public health policies for sustainable, effective PA framework.

## Data Availability

The raw data supporting the conclusion of this article will be made available by the authors, without undue reservation.
