# Supplementary material for: Associations between monitor-independent movement summary (MIMS) and fall risk appraisal combining fear of falling and physiological fall risk in community-dwelling older adults
Source: Front Aging. 2024 Apr 9;5:1284694. doi: 10.3389/fragi.2024.1284694 (PMC11040232; doi:10.3389/fragi.2024.1284694)
Supplement: Supplementary file 1 [file Table1.pdf]

Supplementary Material

Associations between Monitor-Independent Movement Summary (MIMS) and Fall Risk Appraisal Combining Fear of Falling and Physiological Fall Risk in Community-Dwelling Older Adults

Renoa Choudhury [1], Joon-Hyuk Park [1,2*], Chitra Banarjee[3], Miguel Grisales Coca[1], David Fukuda[4], Rui Xie[5], Jeffrey R. Stout[2,4], Ladda Thiamwong[2,6]

*** Correspondence:** Joon-Hyuk Park: joonpark@ucf.edu

Supplementary Table 1: Association between Fall Risk Appraisal groups and average daily MIMS (MIMS/day), controlled by age, gender and BMI using multiple linear regression

Daily MIMS, MIMS/day	*β (SE)*	*p*
Age (years)	-97.63 (32.3)	**.003**
Gender (reference: Female)		
Male	-1080.32 (537.7)	**.046**
BMI (kg/m^2)	-41.83 (44.1)	.344
Fall Risk Appraisal Groups (reference: Rational)		
Irrational	-1603.1 (686.4)	**.021**
Incongruent	-883.9 (539.9)	.104
Congruent	-1206.6 (647.7)	.064
