# Supplementary material for: Associations between monitor-independent movement summary (MIMS) and fall risk appraisal combining fear of falling and physiological fall risk in community-dwelling older adults
Source: Front Aging. 2024 Apr 9;5:1284694. doi: 10.3389/fragi.2024.1284694 (PMC11040232; doi:10.3389/fragi.2024.1284694)
Supplement: Supplementary file 2 [file Table2.pdf]

Supplementary Material

Associations between Monitor-Independent Movement Summary (MIMS) and Fall Risk Appraisal Combining Fear of Falling and Physiological Fall Risk in Community-Dwelling Older Adults

Renoa Choudhury [1], Joon-Hyuk Park [1,2*], Chitra Banarjee[3], Miguel Grisales Coca[1], David Fukuda[4], Rui Xie[5], Jeffrey R. Stout[2,4], Ladda Thiamwong[2,6]

*** Correspondence:** Joon-Hyuk Park: joonpark@ucf.edu

Supplementary Table 2: Association between Fall Risk Appraisal groups and peak 30-minute MIMS per day (MIMS/day), controlled by age, gender and BMI using multiple linear regression

Peak 30-minute MIMS per day, MIMS/day	*β (SE)*	*p*
Age (years)	-0.32 (0.09)	**.001**
Gender (reference: Female)		
Male	-2.15 (1.55)	**.166**
BMI (kg/m^2)	-0.09 (0.13)	.461
Fall Risk Appraisal Groups (reference: Rational)		
Irrational	-5.40 (1.97)	**.007**
Incongruent	-2.52 (1.55)	.106
Congruent	-5.43 (1.86)	**.004**
