# Supplementary material for: Associations between monitor-independent movement summary (MIMS) and fall risk appraisal combining fear of falling and physiological fall risk in community-dwelling older adults
Source: Front Aging. 2024 Apr 9;5:1284694. doi: 10.3389/fragi.2024.1284694 (PMC11040232; doi:10.3389/fragi.2024.1284694)

Associations between Monitor-Independent Movement Summary (MIMS) and Fall Risk Appraisal Combining Fear of Falling and Physiological Fall Risk in Community-Dwelling Older Adults

Renoa Choudhury [1], Joon-Hyuk Park [1,2*], Chitra Banarjee[3], Miguel Grisales Coca[1], David Fukuda[4], Rui Xie[5], Jeffrey R. Stout[2,4], Ladda Thiamwong[2,6]

*** Correspondence:** Joon-Hyuk Park: joonpark@ucf.edu

Supplementary Figure 1. Average age (years) across categories of Fall Risk Appraisal combining fear of falling (FOF) and physiological fall risk, *p < .05, **p < .01.

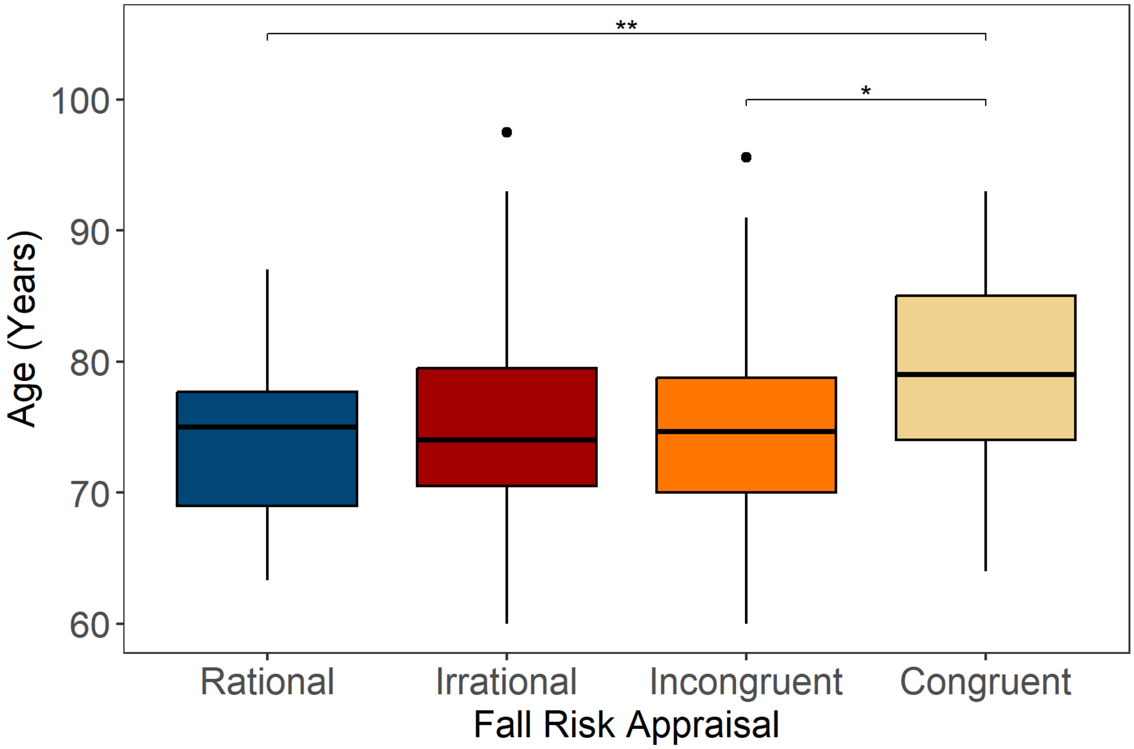

Supplement: Supplementary file 4 [file Image1.pdf]
